# Deafness and the Tokelauan health model

**DOI:** 10.7189/jogh.13.03001

**Published:** 2023-02-24

**Authors:** Latasi Koro, Annette Kaspar

**Affiliations:** 1 Department of Audiology, University of Auckland, Auckland, New Zealand; 2 ENT Department, Tupua Tamasese Meaole Hospital, Ministry of Health, Apia, Samoa; 3 Hearing Research Unit for Children, School of Health and Rehabilitation Sciences, University of Queensland, Brisbane, Australia

**Figure:**
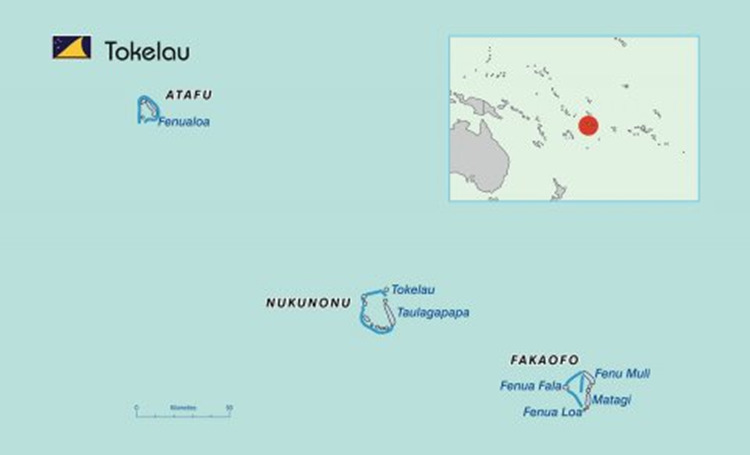
Photo: Map of Tokelau. Source: image freely available from https://sdd.spc.int/tk.

According to the World Health Organization (WHO), the Pacific Island region has among the highest global rates of hearing loss [1]. Ear disease and vaccine-preventable infections are the leading causes of hearing disorders among children [2-4], while non-communicable diseases and age-related hearing difficulties are the major causes among adults [5,6]. Although ear and hearing health professionals are scarce throughout the Pacific Islands [7,8], there are currently efforts under way to address hearing loss through the capacity-development of both clinical and public health service delivery mechanisms [9]. Optimal outcomes may only be achieved through strategies that reflect the local health model and belief systems.

The present viewpoint will illustrate how the Tokelauan health model is a solid foundation for the development of hearing health services in Tokelau, a Polynesian nation of the Pacific Islands [10].

## TOKELAU

Tokelau is a non-self-governing territory of New Zealand, consisting of three coral atolls (illustrated in this viewpoint’s Photo). Classified as a Small Island Developing State, it is accessible only by sea transport from Samoa (24-30 hours). All Tokelauans are New Zealand citizens, with a total population of 1497 (2022 Census, available sdd.spc.int/tk), and a relatively large dependent age group (i.e. young children and older adults). Young adults (25-34 years) tend to migrate overseas to seek education and employment opportunities.

The coral atolls provide a sustainable lifestyle, but this fragile environment is extremely vulnerable to the impacts of climate change. The national development agenda is therefore heavily focused on addressing climate change, and Tokelau aspires to be the first country to use 100% renewable energy.

## TE VAKA ATAFAGA – THE TOKELAUAN HEALTH MODEL

**Figure 1:**
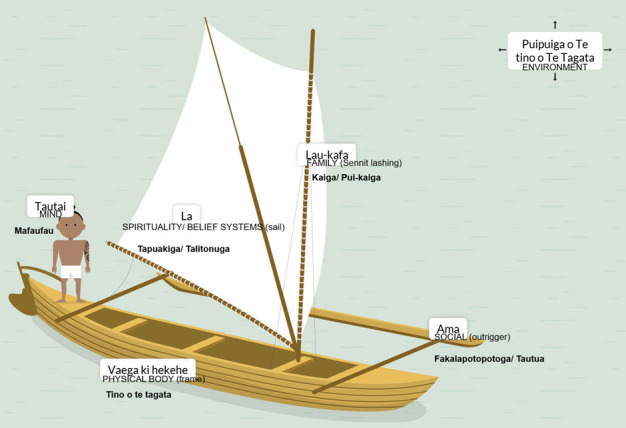
Modified illustration of Tokelauan Model of Health, Te Vaka Atafaga [10].

Te Vaka Atafaga is a published Tokelauan framework that summarises the Tokelauan perception of health and well-being (**Figure 1**) [10]. It is the first and currently the only Tokelauan health model, created by Tokelauan clinician Kupa Kupa, and endorsed by Tokelau elders and community representatives at the Inaugural Tokelau Health National Conference in Wellington, New Zealand 1992 [10].

Te Vaka Atafaga is the Tokelauan word for a traditional outrigger vessel with a sail. Given the importance of the ocean to the Tokelauan way of life, Te Vaka Atafaga illustrates the core beliefs Tokelauans hold regarding health and well-being. Te Vaka Atafaga represents the following six components of the Tokelauan health model as explored below.

### Te tino o te tagata (physical body)

A healthy vessel is essential for a healthy mind and spirit. Age is linked to physical capabilities and associated responsibilities. Infants are considered weak and helpless, and so, are fiercely protected. As children grow and mature, they develop physical capabilities into adulthood. Older adults are then fiercely protected again, as physical strength is seen to decline with age (“kua maui te malohi”).

### Mafaufau (the mind)

The fisherman or expert navigator represents “mafaufau” (the mind), responsible for directing the individual/family/community on a safe and plentiful journey based on their wealth of knowledge and experience. This is the domain of the elders who are held in high regard, and often sought for advice on all matters. “Mafaufau” encapsulates an awareness of how all the different aspects of well-being tie together to benefit self, others and the environment.

### Kaiga (Tokelauan family structure)

The intertwined threads of rope represent “kaiga” (Tokelauan family structure). It illustrates that although individuals have a unique role to play, they are strongly bound through the generations. “Kaiga” tends to be broadly defined, from having a shared or common ancestry, to being part of a universal family through their connection to God. With the Tokelauan diaspora, it recognises the importance of teaching Tokelauan culture and traditions for those in Tokelau, as well as those abroad.

### Tapuakiga/talitonuga (spirituality/belief systems)

The sail represents a notion of Christianity known as “tapuakiga” or “talitonuga” (spirituality/ belief systems). Tui-Tokelau, the ancient paramount symbol of God symbolises unity – unity in distributing resources, care and unconditional love.

### Puipui o te tino o te tagata (environment)

The air represents “puipui o te tino o te tagata” (environment), the physical surrounding of an individual how it influences their well-being. There is a strong sense of connection to the environment, maintained through “fatele” (action composition songs) which tell “kakai” (traditional tales), biblical stories, Tokelauan history, and current events. Indigenous ways of maintaining the land, housing and transportation, and the ability to access or provide adequate resources is considered.

### Fakapotopotoga/tautua (social and support systems)

The outrigger represents “fakapotopotoga/tautua” (social and support systems) which are maintained through church and community groups such as the “aumaga”. These groups embody the Tokelauan value of the “inati” system, where food and other resources are shared equitably among the community, ensuring primarily that those most vulnerable are taken care of (“tamāmanu”).

## DEAFNESS AND THE TOKELAUAN HEALTH MODEL

Audiology is a health profession based on a biomedical model of health care and health service delivery. The evolving body of literature aimed at addressing deafness in low and middle-income countries reflects these biomedical model origins. To achieve optimal hearing health outcomes for populations who adhere to alternative health belief systems, the development of hearing health services must honour local health belief models as their foundation. The discussion below illustrates how the Tokelauan health model is very favourable to the successful implementation of audiology care for Tokelauan populations.

### Holistic care

The Tokelauan health model views health and well-being in a holistic manner. While there is indeed a physical component to deafness (i.e. physiological damage to the auditory system), the mental, spiritual, and collectivist aspects of deafness are considered to be equally important [1]. Hearing health aligns with this worldview given that the adverse impacts of deafness on quality of life are well-known. Although the audiology profession typically concerns itself with the physical aspects (i.e. fitting hearing aids), a successful service for Tokelauans must allow space for the mental, spiritual and social impacts of hearing difficulties to be addressed.

The spiritual component of health care gains further importance when considering that the well-being of an individual is viewed as collective responsibility (“inati”). When hearing health care professionals acknowledge spirituality, they are stating that although hearing solutions may be at the physical level (i.e. improvement in hearing levels of the individual), the benefits of hearing health are for both individuals and the collective community. This supports what is already known within the audiology profession, that hearing difficulties impact not only the person who is deaf or hard of Hearing, but extends to their family and loved ones. “Te Vaka Atafaga” promotes this aspect of hearing health care perfectly.

### Community-based care

The intertwined family structure offers an ideal framework for community-based health care delivery. The wisdom of elders is usually sought for any activities that impact family life, and the raising of children is considered the responsibility of all. Community-based care for hearing health should first involve education on the role of each family member in caring for the person with hearing impairment, whether it is helping an older person manage their hearing aids, or performing ear mopping for a child with a severe ear infection. Health education and messaging should align with support systems, and highlight Tokelauan narratives of language, family and culture to promote better community understanding of hearing loss and its impact. Hearing health advocates may then also facilitate community-led solutions to improving listening skills for all during important gatherings such as church or community meetings. Given that social connection is what is most important for Tokelauans, leaning into health promotion or prevention measures that promote social connectedness would be most effective.

### Spirituality

Spirituality should be considered during health promotion activities. A significant proportion of avoidable hearing difficulties may be addressed through health promotion [9], and combining biblical/spiritual teaching with health education is potentially very powerful. For example, “Psalms 139 You are fearfully and wonderfully made” can support the practical recommendations that “burst eardrums will heal on their own, but the ear must be kept clear and dry” or “you don’t need to use earbuds because your ears clean themselves”. Audiologists are often asked if a person with hearing difficulties in both ears should use one hearing aid or two, and under the Tokelauan health model it is perfectly appropriate to answer “God created us with two ears for a reason, so the best advice is two hearing aids”.

### Physical environment

From a practical perspective, the role of the environment applies to hearing health initiatives in the sense that any health initiative must be locally sustainable given the remote geographical location of Tokelau. Initiatives must involve community leadership and the capacity-development of local health workers. The strong emphasis on collective well-being aligns with public health messaging for hearing health, with the promotion of healthier environments benefitting the health of all (i.e. smoke-free, safe noise levels).

### Interconnectedness

The Tokelauan health model is inherently about interconnectedness. Addressing hearing loss in Tokelau has many aspects that are fundamentally interconnected. The delivery of hearing health care, whether in the context of individual clinical care or at the public health level, must reflect its connection to holistic health, and its benefits for both the individual and the community.

## CONCLUSION

Although a specialist field, hearing health care is a profession that lends itself well to the Tokelauan health model which holds a holistic worldview of interconnectedness. Although the present viewpoint uses audiology to illustrate the role of this model in the development of health services, the principles described should be transferable to all areas of health, as well as other Pacific Island cultures.
